# Passive carriage of rabies virus by dendritic cells

**DOI:** 10.1186/2193-1801-2-419

**Published:** 2013-08-29

**Authors:** Kazuyo Senba, Takashi Matsumoto, Kentaro Yamada, Seiji Shiota, Hidekatsu Iha, Yukari Date, Motoaki Ohtsubo, Akira Nishizono

**Affiliations:** Department of Microbiology, Faculty of Medicine, Oita University, 1-1 Idaigaoka, Hasama-machi, Yufu-City, Oita, 879-5593 Japan; Faculty of Food Science and Nutrition, Beppu University, Beppu, Oita, Japan; Frontier Science Research Center, Faculty of Medicine, Miyazaki University, Miyazaki, Japan

**Keywords:** Rabies virus, Dendritic cells, Immune invasion

## Abstract

The rabies virus (RABV) is highly neurotropic and it uses evasive strategies to successfully evade the host immune system. Because rabies is often fatal, understanding the basic processes of the virus-host interactions, particularly in the initial events of infection, is critical for the design of new therapeutic approaches to target RABV. Here, we examined the possible role of dendritic cells (DCs) in the transmission of RABV to neural cells at peripheral site of exposure. Viral replication only occurred at a low level in the DC cell line, JAWS II, after its infection with either pathogenic RABV (CVS strain) or low-pathogenic RABV (ERA strain), and no progeny viruses were produced in the culture supernatants. However, both viral genomic RNAs were retained in the long term after infection and maintained their infectivity. The biggest difference between CVS and ERA was in their ability to induce type I interferons. Although the ERA-infected JAWS II cells exhibited cytopathic effect and were apparently killed by normal spleen cells *in vitro*, the CVS-infected JAWS II cells showed milder cytopathic effect and less lysis when cocultured with spleen cells. Strongly increased expression of major histocompatibility complex classes I, costimulatory molecules (CD80 and CD86), type I interferons and Toll- like receptor 3, and was observed only in the ERA-inoculated JAWS II cells and not in those inoculated with CVS. During the silencing of the cellular immune response in the DCs, the pathogenic CVS strain cryptically maintained an infectious viral genome and was capable of transmitting infectious RABV to permissive neural cells. These findings demonstrate that DCs may play a role in the passive carriage of RABV during natural rabies infections.

**Electronic supplementary material:**

The online version of this article (doi:10.1186/2193-1801-2-419) contains supplementary material, which is available to authorized users.

## Background

Innate immunity is the first line of defense against invading pathogens and encompasses several reactions that target the initial phases of viral infections. Dendritic cells (DCs) are the most efficient type of antigen-presenting cell and play a pivotal role in orchestrating both the innate and adaptive immune responses during viral infections (Steinman 1991). Upon the uptake of certain viral antigens, DCs undergo maturation, which involves the up-regulation of surface major histocompatibility complex (MHC) molecules and the production of type I interferon (IFN), including IFN-α and –β (Barchet et al. 2002; Cheng et al. 2004; Dhib-Jalbut and Cowan 1993). During viral infection, the activation of retinoic-acid-inducible gene I protein (RIG1), a cellular DExD/H box RNA helicase that induces the production of antiviral factors, following viral RNA detection, leads to increased type I IFN production (Hornung et al. 2006; Imaizumi et al. 2004; Rothenfusser et al. 2005; Sakaki et al. 2005). However, several viruses tend to elicit only weak immune responses or immune responses that are insufficient to eliminate the invading pathogen, and it has been suggested that certain RNA viruses specifically target RIG1 to allow immune evasion (Leung et al. 2012).

Rabies, although not preeminent among current infectious diseases, continues to afflict humans, with as many as 55,000 deaths annually. The fatality rate remains the highest among infectious diseases, and medical treatments have proven ineffective (Jackson 2013). Rabies virus (RABV) is a highly neurotropic negative-stranded RNA virus that spreads along the neural pathway and invades the central nervous system (CNS), where it causes an acute and often fatal infection (Finke and Conzelmann 2005). One of the characteristic features of RABV is its ability to reach the CNS in spite of inducing an immune response that can eliminate the virus (Feder et al. 2012). This ability is considered particularly important during the initial stages of peripheral infection following an animal bite, the most common route of transmission, as the virus moves from the muscle or subcutaneous tissue to the peripheral neuronal cells. Although reaching the CNS represents a critical stage in the establishment of the infection, the pathogenesis and detailed mechanism of host evasion and the preservation of RABV during these early stages are not well understood.

Several types of cells directly interact with RABV in the early stages of infection, at the site of initial exposure. A recent study has shown that vesicular stomatitis virus, which belongs to the same genus *Rhabdoviridae*, gains access to the CNS through the peripheral nerves in macrophage-depleted lymph nodes (Iannacone et al. 2010). RABV particles that are injected into the skin or muscles can slowly replicate in these tissues before they reach the CNS (Charlton et al. 1997; Murphy et al. 1973). It is unclear whether RABV directly invades neural cells or is sequestered by nonpermissive cells that subsequently migrate to neural cells. Recently, Li et al. demonstrated that infection with a pathogenic strain of RABV led to weak DC maturation, whereas a low-pathogenic strain caused the up-regulation of the NF- κB signaling pathway, resulting in high levels of IFN-α mRNA in immature DCs (Li et al. 2008). RABVs are generally classified into two categories: field-isolated virus (street viruses) and laboratory-adapted viruses (fixed strains). The former are known to be more pathogenic after peripheral infections and highly neuropathogenic. In contrast, the latter generally lose their infectivity after peripheral inoculation, despite the preservation of their neuropathogenicity (Lepine 1938). Among the fixed RABVs, representative low-pathogenic strains, including Evelyn-Rokitnicki-Abelseth (ERA), Street Alabama Dufferin and Vnukovo-32, also trigger apoptosis in both neural and nonneural cells, which acts as a potent activator of the immune response, leading to the effective elimination of RABV from the CNS (Préhaud et al. 2003; Thoulouze et al. 1997). In contrast, pathogenic RABV strains, such as the challenge virus standard (CVS), are postulated to evade the host immune surveillance system and to reach neural cells of the CNS by two different mechanisms (Lafon 2011; Rieder and Conzelmann 2011). Here, we hypothesize that immature DCs harboring infectious viruses are present for a long time at the site of initial exposure to RABV. In this study, we sought to clarify the possible role of RABV-infected DCs at the initial infection site in the early phase of RABV infection.

## Results

### Susceptibility of JAWS II cells to RABV

**Figure 1:**
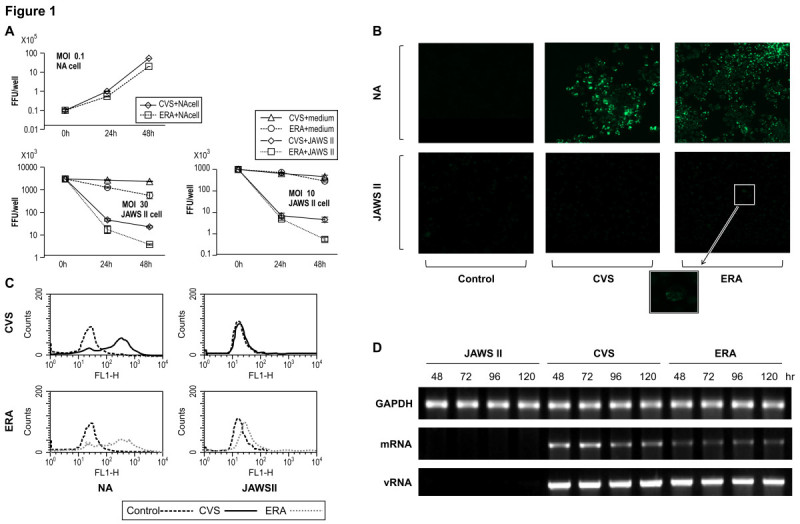
**Virus growth in JAWS II and NA cells inoculated with rabies virus. (A)** Virus growth curves in NA and JAWS II cells. CVS or ERA was infected at an MOI of 0.1 (NA) or 10,30 (JAWS II), and samples were collected 24 and 48 h after infection. n = 3. Error bars represent mean ± SEM. The black line and dotted line represent the viral titers of CVS and ERA, respectively. Decline in the infected viral titer was determined by using JAWS II cells (+JAWS II) and cell-free conditions (+medium). **(B and C)** The expression of the viral N protein was analyzed with both a laser scanning microscope and a flow cytometer. After the infection of JAWS II cells with CVS or ERA, the cells were permeabilized with IntraPrep Permeabilization Reagent, and then incubated with FITC-conjugated anti-RABV N MAb. **(D)** Detection of mRNA and genomic RNA in CVS- or ERA-infected JAWS II cells. The upper panel shows GAPDH, the middle panel shows the expression of mRNA from the RABV N gene detected by RT–PCR, using a poly (T)_15_ primer for the synthesis of the first-strand cDNA and the lower panel shows viral RNA. The experiments were performed three times and representative data are shown. CVS or ERA at an MOI of 10 was used for the experiments shown in **(B to D)**.

Previous studies have demonstrated that several pathogenic and non-pathogenic RABV strains can infect immune-related cells, including T cells and monocytes (Thoulouze et al. 1997; Nakamichi et al. 2004). Therefore, we examined whether the DC line, JAWS II, is susceptible to RABV infection using two types of RABV strains; pathogenic CVS and low -pathogenic ERA. We first examined whether RABV can replicate in JAWS II cells and produce progeny viruses in the culture supernatant. The growth kinetics of CVS and ERA in NA cells was determined and both RABV strains replicated significantly at an MOI of 0.1; at 48 h after infection, their titers were in the range of 10^6^ ~10^7^ ffu/well (Figure 1A). However, both CVS and ERA viral titers declined rapidly and neither had effectively replicated in the JAWS II cells at 48 h after infection, even at a high MOI of 10 or 30 (Figure 1A). Interestingly, infectious RABV at an MOI of 10 or 30 gradually decreased under cell-free conditions (CVS or ERA + medium, in Figure 1A) as a natural result because the virus could not survive without infection into the cells, although a more rapid decline in infectivity was observed in the presence of JAWS II cells (CVS or ERA + JAWS II, in Figure 1A), indicating that RABV was promptly ingested by the cells and that no infectious virus remained in the culture medium.

Because RABV N protein is the most abundantly produced viral protein in infected cells, we examined the expression of RABV N protein in the JAWS II cells with laser scanning microscopy and FACSCalibur flow cytometry. Both CVS and ERA effectively propagated in the cells and strong expression of N protein was observed in the NA cells with fluorescence-activated cell sorting (FACS) and immunofluorescence analyses (Figure 1B and C). In contrast, no fluorescence activity was observed in the CVS-infected JAWS II cells (Figure 1B and C), and although a mild increase in fluorescence intensity was observed in the ERA-infected JAWS II cells (Figure 1B and C). Based on these observations, the JAWS II cells were slightly permissive to ERA infection, but were not susceptible to CVS infection.

Following the experiments described above, in which the cells were infected with CVS or ERA, we attempted to detect mRNA and viral genomic RNA in the cells using RT-PCR at 48, 72, 96, and 120 h after infection. Low levels of mRNA signals were detected in both the CVS- and ERA-infected JAWS II cells within 48 h (Figure 1D). Interestingly, viral genomic RNA was sustainably detected in the JAWS II cells infected with either CVS or ERA, even after 120 h (Figure 1D).

### Cell death of ERA-infected JAWS II cells induced by viral infection and coculture with spleen cells

**Figure 2:**
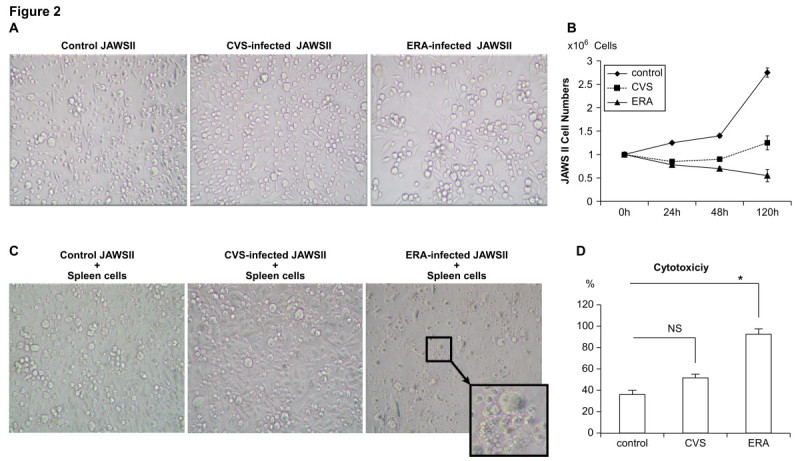
**Morphological changes in JAWS II cells after RABV infection and coculture with mouse spleen cells. (A)** Light-microscopic images of uninfected (left), CVS-infected (middle), and ERA-infected (right) JAWS II cells; **(B)** the numbers of viable cells were determined by Trypan blue staining 24, 48, 120 h after re-plated. **(C and D)** uninfected (left), CVS-infected (middle), and ERA-infected (right) JAWS II cells were cocultured with mouse spleen cells for a further 120 h and the cytotoxicity was calculated with a cell death kit. All the experiments were performed three times at an MOI of 10 and representative data are shown **(A and C)**. Error bars represent mean ± SEM **(B)**. *p < 0.01 **(D)**.

Previous studies have demonstrated that low -pathogenic RABV induces apoptosis in neural and nonneural cells (Préhaud et al. 2003; Thoulouze et al. 1997). ERA infected JAWS II cells at 120 h after re-plated to a new plate had decreased significantly (by nearly 80%), whereas that the number of CVS-infected JAWS II cells was slightly increased (Figure 2A). Light microscopic observation showed that the ERA-infected JAWS II cells had a relatively high cytopathic effect compared with the CVS-infected JAWS II cells (Figure 2A and B). Using a coculture assay, we examined whether RABV-infected JAWS II cells were recognized by and lysed in the presence of spleen cells. Normal spleen cells cocultured with uninfected JAWS II cells showed spontaneous cytotoxicity of 36 ± 4.03%. After infection with ERA, JAWS II cells showed ballooning, fragmentation, or condensation of their cytoplasm (Figure 2C and D). The ERA-infected JAWS II cells showed significantly higher cytotoxicity (92.5 ± 6.02%) than the CVS-infected JAWS II cells.

### Levels of MHC class I and II molecules costimulatory molecules, innate-immunity-related molecules, and the production of type I IFNs elevated in ERA-infected JAWS II cells

**Figure 3:**
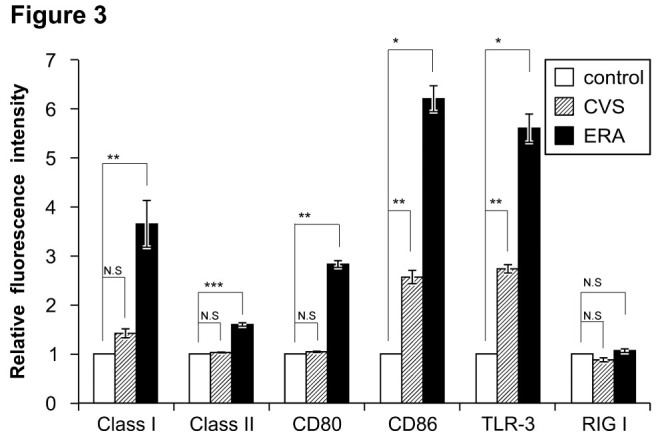
**Expression of cell-surface molecules by flowcytometric analysis at 48 h after infection of CVS or ERA (MOI of 10).** Profiles of the surface molecules on uninfected (control; white), CVS-infected (gray), and ERA-infected (black) JAWS II cells reacted with FITC conjugated anti-mouse MHC class I, MHC class II and CD80, PE conjugated anti-mouse CD86 and TLR3, and rat anti-mouse RIG1 antibodies, followed by a FITC-conjugated goat anti-rat IgG antibody. The relative fluorescence intensity of the surface molecules was determined with FACS. All cells including uninfected cells (control) were stained by the same procedure. The experiments were performed three times and error bars represent SEM. *p < 0.001, **p < 0.05, ***p < 0.01.

To determine whether CVS-infected JAWS II cells can avoid eliciting host immune responses, we examined the immune-related surface molecules on JAWS II cells at 48 h after their infection with CVS or ERA at an MOI of 10. Because MHC classes I and II are antigen-presenting molecules important in the activation of the immune responses, their expression on the surfaces of JAWS II cells was examined by flow cytometry. The analysis revealed that the expression of MHC class I increased more strongly on the surfaces of the cells infected with ERA (Figure 3). Similarly, the expression of the costimulatory molecules, CD80 and CD86 was significantly more strongly upregulated in the ERA-infected JAWS II cells than in the CVS-infected JAWS II cells.

**Figure 4:**
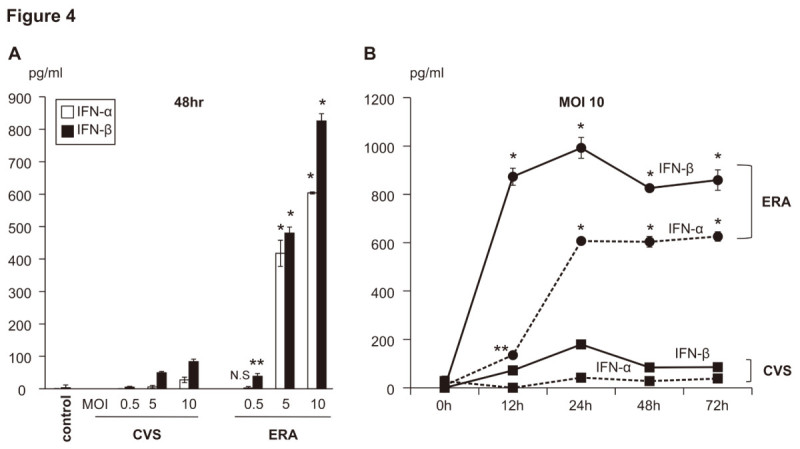
**Production of IFN-α and IFN-β in CVS- and ERA-inoculated JAWS II cells. (A)** The levels of IFN-α (white) and IFN-β (black) in the culture supernatant 48 h after infection in CVS- or ERA-infected JAWS II cells at various MOIs (0.5, 5, and 10). **(B)** Serial changes in IFN-α (dotted line) and IFN-β (black line) production in the CVS-infected (squares) and ERA-infected JAWS II cells (circles) at an MOI of 10. The levels of type I IFN were quantified by ELISA 72 h after infection. The experiments were performed three times and error bars represent SEM. *p < 0.001, **p < 0.01.

The phosphoprotein encoded by RABV is known to possess an antagonizing activity against the induction of IFN by RIG1 in host cells (Brzózka et al. 2006). Toll-like receptors (TLRs)-3, -7, -8, and −9 act as sensors of viral nucleic acids and, with the exception of the TLR-3 signaling pathway, also induce inflammatory cytokines and IFNs. Therefore, we also examined how innate immune sensor molecules are expressed in RABV-inoculated DCs, using flow cytometry. The expression of TLR-3 was more strongly elevated in the ERA-infected JAWS II cells than in the CVS-infected JAWS II cells, when examined flow cytometry (Figure 3). It is well known that the type I IFN response controls tissue tropism and pathogenicity during viral infection processes (Chopy et al. 2011). As well as analyzing the expression of MHC and costimulatory molecules after RABV infection, we examined the production of the type I IFNs, IFN-α and -β, in RABV-infected DCs using an ELISA. We determined that the production of IFN-α and -β was markedly higher in ERA-infected JAWS II cells than in cells infected with CVS (Figure 4).

### Possible cell-to-cell infection by RABV transmitted from JAWS II cells to neural cells

**Figure 5:**
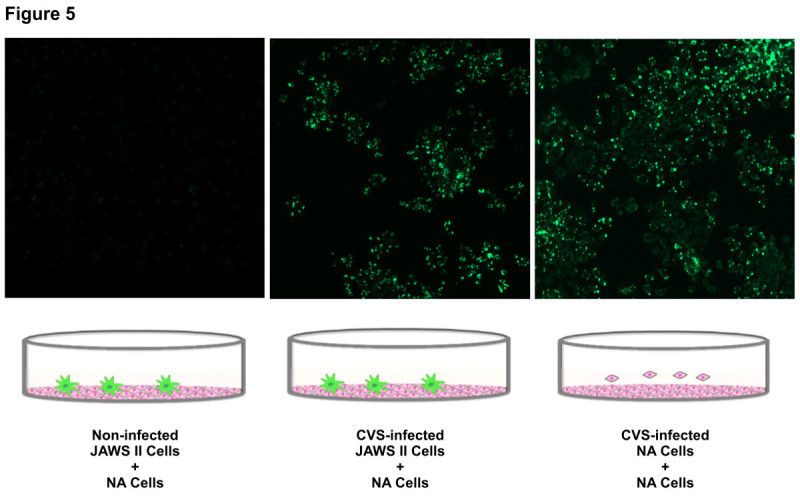
**Cell-to-cell infection of RABV from JAWS II cells to neural cells.** A coculture assay of NA cells (indicator cells) with CVS-infected JAWS II cells or NA cells (donor cells) was performed as described in the Methods. The expression of the viral N protein was analyzed with a laser scanning microscope. After the infection of JAWS II cells with CVS at an MOI of 10, the cells were permeabilized with IntraPrep Permeabilization Reagent, and then reacted with FITC-conjugated anti-RABV N MAb. The experiments were performed three times and representative data are shown.

To confirm the hypothesis that DCs carry RABV and can cryptically transmit RABV to neural cells, we performed *in vitro* coculture assays to determine whether JAWS II cells harboring the CVS genome facilitate viral transmission *via* cell-to-cell infection. Neither productive viral replication nor viral antigen expression was demonstrated in the CVS-infected JAWS II cells in the analyses described above (Figure 1). Although CVS-infected JAWS II cells did not exhibit progeny viral production when assayed at the protein or genomic level, they did transmit “infectious viral genomes” to uninfected “naïve” NA cells, indicating the occurrence of cell-to-cell transmission (Figure 5). RABV easily replicated and produced progeny virus in the NA cells, so the CVS-infected NA cells were capable of transmitting cell-free virus or cell-associated virus (Figure 5). These results also indicate that the CVS genome was maintained in the JAWS II cells at detectable levels but avoided the host immune system, because it did not induce type I IFNs or upregulate the expression of MHC class I molecule. However, it retained the ability to infect neural cells *in vitro* through the process of cell-to-cell or “*trans*” infection.

### Transmission of RABV to mouse brain *via* JAWS II cells harboring RABV

**Figure 6:**
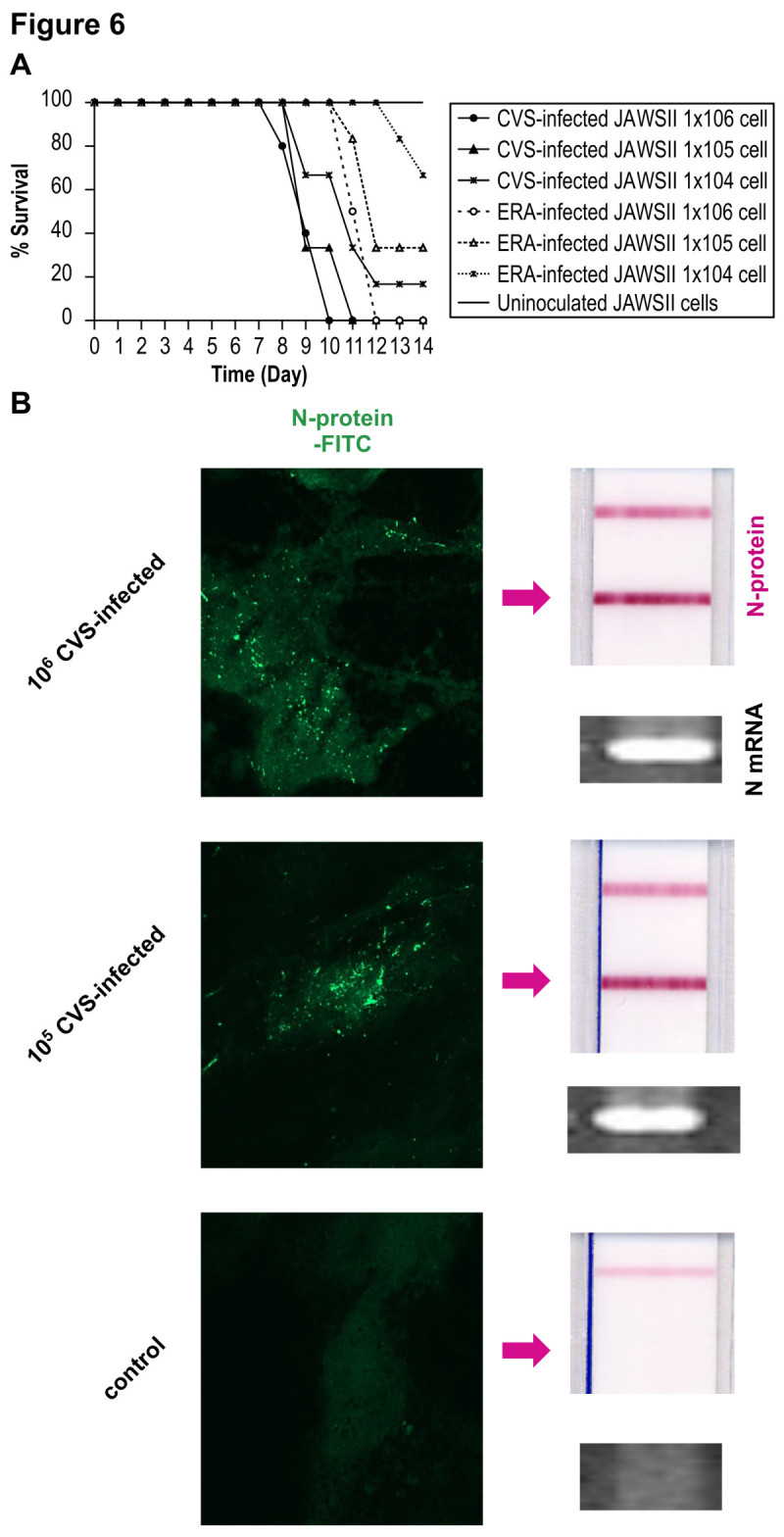
**Transmission of RABV to mouse brain*via*JAWS II cells harboring RABV. (A)** Survival rates of five-week-old C57BL/6 mice injected intracerebrally with 10^6^, 10^5^, or 10^4^ CVS- or ERA-infected JAWS II cells (n = 10 each group) at MOI of 30. Their survival was observed for 14 days after the transfer of the cells. The black and dotted lines represent the survival of the mice injected with CVS- and ERA-infected JAWS II cells, respectively. The circles indicate the survival of mice injected with 10^6^ CVS- or ERA-infected JAWS II cells. The triangles indicate the survival of mice injected with 10^5^ CVS- or ERA-infected JAWS II cells. The crosses indicate the survival of mice injected with 10^4^ CVS- or ERA-inoculated JAWS II cells. The experiments were performed three times. The Kaplan-Meier method was used to analyze mouse survival. Statistical analyses were performed by log-rank test. (p < 0.01; 10^6^ CVS: 10^6^ ERA, 10^5^ CVS: 10^5^ ERA. p <0.05; 10^4^ CVS: 10^4^ ERA) **(B)** Half the mice from each group were sacrificed after seven days, and the presence of viral N mRNA and RABV N protein in the hippocampal tissues was determined by RT–PCR (mRNA) and a RABV N detection kit with FITC staining, respectively. The arrow indicates the positive band of N protein on the immunochromatographic test. The upper band corresponds to nonspecific control. The experiments were performed three times as shown in Figure 1B and representative pictures are shown.

To examine whether cell-to-cell transmission occurs from DCs to neural cells *in vivo*, we directly examined the effects of the passive transfer of RABV-infected DCs to mice. Five-week-old C57BL/6 mice were injected intracerebrally with 10^6^, 10^5^, or 10^4^ CVS- or ERA-infected JAWS II cells (n = 10 each group), and their survival was monitored for 14 days after transfer. No deaths were observed in the control group injected with RABV-uninfected DCs. Mice injected with either CVS- or ERA-infected JAWS II cells showed clinical symptoms and finally died in a dose-dependent manner (Figure 6A). However, the survival of the mice injected with CVS- or ERA-infected JAWS II cells differed. All mice injected with 10^6^ CVS-infected JAWS II cells died within 10 days, whereas those injected with 10^6^ ERA-infected JAWS II cells survived two days longer. Mice injected with 10^4^ CVS-infected JAWS II cells had a survival rate of only 20%, whereas 70% survival was observed in mice injected with same number of ERA-infected JAWS II cells (Figure 6A). The mice injected with ERA-infected JAWS II cells showed less lethality and longer survival than those injected with CVS-infected JAWS II cells, indicating that ERA-infected JAWS II cells successfully induced the optimal immune responses required for viral elimination *in vivo* (Figure 6A). The surviving mice showed no apparent neurological manifestations or sequelae during the observation period. To confirm viral propagation in the mouse brain, the presence of mRNA and N protein was examined in the hippocampal tissues from mice injected with CVS-infected JAWS II cells. Viral mRNA was clearly detected by RT–PCR and viral N protein was confirmed with laser scanning microscopy and an immunochromatographic test (Figure 6B).

## Discussion

To examine the potential role of DCs in the transmission of RABV, we focused on the probable events that occur at the site of injury caused by rabid animals, by looking into the migration of RABV toward the CNS, which is the organ most permissive to RABV. Neither pathogenic CVS nor low-pathogenic ERA actively produced progeny viruses in JAWS II cells, which were used in this study. However, viral genomic RNA lodged persistently inside the virus-infected cells and was still infectious. Based on our observations, we propose two possible explanations of how RABV evades the host’s immune surveillance system and finally establishes a CNS infection, after which it reaches the brain cryptically. One possibility is that pathogenic RABV does not induce effective host responses for viral elimination in infected DCs; and the other is that the RABV hidden in these DCs plays a role in its cell-to-cell or *trans* infection, thus using DCs as a vehicle in the infection pathway.

In the early phase of the mediation between the innate and acquired immune responses, DCs predominantly reside in the peripheral tissues and play a role as sentinel cells in antigen capture. Immature DCs undergo maturation, characterized by the upregulation of surface MHC molecules and costimulatory molecules, and the subsequent release of numerous humoral factors, including cytokines and IFNs. Subsequently, the mature DCs migrate to the peripheral secondary lymphoid tissues, resulting in the presentation of optimally processed antigen to T lymphocytes *via* these immune synapses. Our flowcytometric and ELISA analyses revealed that the upregulated expression of MHC I molecule on the surface of JAWS II cells and the secretion of type I IFNs were much greater after they were infected with the low-pathogenic RABV strain, ERA, than when they were infected with the pathogenic CVS strain. Analyses of the surface immune molecules revealed that the JAWS II cells matured from the immature state after infection with ERA, but not after infection with CVS.

The mechanism through which JAWS II cells, which are nonpermissive to RABV, can induce this immunological maturation is explained as follows. The small amount of N protein produced in the ERA-infected JAWS II cells (Figure 1B and C) might not be utilized for viral production or morphogenesis, but may be degraded in cellular proteasome and finally assembled with MHC class I molecules, or the minimal N protein produced ERA may be presented directly on MHC class I molecules *via* a cross-presentation process. Another possibility is that in response to certain inhibitory molecules (eg. microRNAs) that are only produced during CVS infection, type I IFNs are not induced in the CVS-infected JAWS II cells (Zhao et al. 2012).

The inadequate immune response stimulated by CVS is also supported by the observation that the death of CVS-infected JAWS II cells was not induced in the presence of naïve spleen cells, whereas the ERA-infected cells were successfully lysed. Although we could not confirm that this was the result of apoptosis, a previous study has demonstrated that low-pathogenic strains of RABV can induce apoptotic responses in macrophages (Jackson and Rossiter 1997). Therefore, it appears that the pathogenic RABV remains hidden within DCs, where it effectively evades the host’s immune surveillance system and may prevent the migration of the infected DCs to the lymphoid tissues. Since we were not able to detect infectious viruses in supernatants of infected JAWS II cells, it is also possible that the rate of virus degradation and re-adsorption is higher than the rate of virus release.

The possibility that RABV exists within DCs below the detection limit of the immune surveillance system has important implications for the spread of RABV from the initial site of infection to the peripheral secondary lymphoid tissues and neural cells, and for the fate of RABV during the long-term incubation period that is often observed after the initial infection (Smith et al. 1991). Although the JAWS II cells were nonpermissive for RABV replication and could not produce progeny virus, *in vitro* coculture studies revealed that NA cells allowed viral transmission after direct cell-to-cell contact with CVS–infected JAWS II cells. This phenomenon is supported by the fact that the exposure of CVS- infected JAWS II cells to monoclonal antibodies directed against a RABV surface glycoprotein (G) 100% blocked the transmission of RABV to NA (KS and AN, unpublished observation). The relevance of viral transmission by a cell-to-cell contact process was demonstrated by the injection of CVS-infected JAWS II cells into the brains of mice, which led to the rapid spread of the virus and 100% mortality. In the establishment of immunodeficiency virus 1 (HIV-1) infection, the uptake of HIV-1 by immature DCs at the mucosal site occurs when it binds to either DC-SIGN or syndecan-3 (de Witte et al. 2007; Geijtenbeek et al. 2000). Following the migration of HIV-1 from the initial mucosal invasion site, DCs may carry the virus to the draining lymph nodes. It has been demonstrated that during the antigen presentation process, DCs cluster with T cells in the secondary lymphoid tissues (de Witte et al. 2007; Geijtenbeek et al. 2000; Lore and Larsson 2003). It is well known that abundant nerve-end fibers innervate the peripheral lymphoid tissue. Other studies have suggested that prions initially accumulate on follicular dendritic cells in the lymphoid tissues and subsequently spread *via* the peripheral nervous system to the brain (Raymond and Mabbott 2007; Sethi et al. 2007). Recently, it has been shown that micro RNAs secreted by Epstein-Barr virus-infected cells are transferred to uninfected cells *via* exosome structures (Pegtel et al. 2010). Our results also suggest that a mechanism similar to that discussed above is involved in the transmission of RABV from DCs to CNS.

DCs are heterogeneous cells and comprise several subsets characterized by unique morphological shapes and functions. Conventional or myeloid-type DCs (mDCs) have a dendritic shape, exhibit typical DC functions, such as antigen uptake, processing, and presentation, and are characterized as CD11c+, CD11b+, and B220-. In contrast, plasmacytoid DCs, which are nondendritic round cells, are defined as CD11^low^, CD11b^-^, and B220^+^ cells, and lack the ability to produce costimulatory molecules (Siegemund et al. 2009). Whereas mDC subsets migrate to the lymph nodes from peripheral tissue, plasmacytoid DCs directly enter the lymph nodes from the blood by crossing high endothelial venules (HEVs) (Banchereau and Steinman 1998; Shortman and Liu 2002) and are able to transform into mDCs in response to the surrounding conditions (Fukao et al. 2000; Fukao et al. 2001; Zuniga et al. 2004). When the cell-surface markers on JAWS II cells were analyzed by flow cytometry, CD11c and CD11b were detected, whereas CD8α was not, and the expression of costimulatory molecules was upregulated by stimulation with viral antigens. These results indicate that JAWS II cells are mDCs, which is consistent with a previous report (Otsu et al. 2006). It has also been speculated that the administration of RABV vaccine *via* an intradermal route in humans, as well as *via* the routine intramuscular route, induce adequate levels of viral neutralizing antibody (Shiota et al. 2008), indicating that mDC subset are recruited to the vaccination site and migrate to the lymph nodes for effective antigen presentation to naïve T cells. Therefore, we speculate that mDCs may play a critical role in RABV infection and pathogenesis.

Several limitations of our study warrant mention. Our attempts to determine whether RABV particles could be visualized in CVS-infected JAWS II cells by immunogold electron microscopy were unsuccessful. We were also unable to confirm the occurrence of apoptosis in the presence of spleen lymphocytes. However, high level of IFN was detected, indicating that cellular immunity was fully functional after the infection of JAWS II cells with ERA.

We have provided several lines of evidence that DCs allow RABV to escape the host immune surveillance system and facilitate the transmission of the virus to the neural cells. Following the uptake of pathogenic CVS, immature DCs do not properly process the viral antigens, as indicated by their lack of MHC class I and II expression. Because the RABV genome persists undetected in DCs and does not replicate or produce progeny virus, we propose that RABV uses these nonpermissive cells as a vehicle to reach the peripheral neural cells, where the virus can then replicate and spread to the CNS. This is the first report to demonstrate that DCs can transmit RABV to neural cells by cell-to-cell or *trans* infection, and sheds new light on the immune evasion strategies used by this deadly virus.

## Methods

### Cells

The DC cell line, JAWS II, and neuroblastoma (NA) cells were purchased from the American Type Culture Collection (Manassas, VA). JAWS II cells (CD11b+, CD11c+, CD8α-, TLR7+, and RIG-1+) were originally isolated from bone-marrow cultures from p53-deficient C57BL/6 mice. The cells were grown in a complete culture medium consisting of RPMI 1640 medium with GlutaMAX™ (Gibco BRL, Grand Island, NY) and supplemented with 10% fetal bovine serum (FBS), 1% penicillin-streptomycin, 50 μm 2-mercaptoethanol, and 5 ng/mL recombinant mouse granulocyte-macrophage colony-stimulating factor (BD Biosciences, San Jose, CA). Murine NA cells were grown in Eagle’s minimal essential medium (Gibco BRL, Grand Island, NY) containing 10% FBS and 1% penicillin-streptomycin.

### Viruses

A pathogenic strain of RABV (CVS-11) and a low- pathogenic strain (ERA) were propagated in NA cells. The viruses in the culture supernatants of the RABV-inoculated NA cells were collected and centrifuged at 1200 × *g* for 10 min at 4°C (TOMY EX-125, TS-38, TOMY, Tokyo, Japan), and then purified by ultrafiltration through a 0.45-μm filter. The infection of NA or JAWS II cells with RABV was performed at 37°C at various multiplicities of infection (MOIs).

### Antibodies

Fluorescein isothiocyanate (FITC)-conjugated goat anti-rat IgG antibody was obtained from Organon Teknika Corp. (Durham, NC). FITC-conjugated anti-mouse MHC class I, MHC class II, and CD80 antibodies, and phycoerythrin (PE)-conjugated anti-mouse CD86 antibody were purchased from eBioscience Inc. (San Diego, CA). PE-conjugated anti-mouse CD283 (TLR3) antibody and purified rat anti RIG1 antibody were obtained from BioLegend Inc. (San Diego, CA). FITC-conjugated anti-RABV N monoclonal antibody (MAb) was obtained from Fujirebio Diagnostics, Inc. (Malvern, PA).

### Measurement of viral replication

NA and JAWS II cells were seeded in 48-well culture plates (10^5^ cells/well) and infected with CVS or ERA at the appropriate MOI. The culture supernatants obtained from the RABV-infected NA or JAWS II cells were then separated by centrifugation at 1200 × *g* for 10 min 0, 24, or 48 h after infection. The virions in the supernatant were purified by ultrafiltration through a 0.45 μm filter and then titrated. The viruses in the culture supernatants were titrated with a focus assay on confluent monolayers of NA cells in 24-well plates, according to previously reported procedures (Yamada et al. 2012).

### Flow cytometry and confocal analysis

The expression of cell-surface molecules was measured by immunofluorescence flow-cytometric analysis using a FACSCalibur flow cytometer (BD Immunocytometry Systems, San Jose, CA) and CellQuest software. For the analysis, NA and JAWS II cells were first seeded in six-well culture plates (10^6^ cells/well) and infected with CVS or ERA at an MOI of 10. After 48 h at 37°C, the cells were collected and washed in phosphate-buffered saline (PBS), and then incubated on ice for 30 min with a FITC-conjugated anti-mouse MHC class I (1:50), MHC class II (1:50), or CD80 (1:50) antibody, or PE-conjugated anti-mouse CD86 antibody (1:50). The expression of the intracellular proteins, TLR-3 and RIG1 was also measured by immunofluorescence flow-cytometric analysis using a FACSCalibur flow cytometer and CellQuest software. After the infection of JAWS II cells with CVS or ERA (as described above), the cells were permeabilized with Intra Prep Permeabilization Reagent (Immunotech, Marseille Cedex, France), according to the manufacturer’s protocol, and then reacted with PE-conjugated anti-mouse TLR3 (1:50) or rat anti-mouse RIG-1 (1:50) antibody. An FITC-conjugated goat anti-rat IgG (1:200) was used as the secondary antibody to detected RIG1.

The expression of the viral N protein was analyzed with both a laser scanning microscope (Carl Zeiss MicroImaging, LSM 510; Carl Zeiss, Jena, Germany) and a FACSCalibur flow cytometer. After the infection of JAWS II cells with CVS or ERA (as described above), the cells were permeabilized with IntraPrep Permeabilization Reagent, and then reacted with FITC-conjugated anti-RV N MAb (1:50).

### Enzyme-linked immunosorbent assays (ELISAs)

To measure the quantities of IFN-α and IFN-β secreted by the RABV-infected JAWS II cells, JAWS II cells seeded in six-well plates (10^6^ cells/well) were inoculated with CVS or ERA at an MOI of 10 and the culture supernatants were then harvested at 0, 12, 24, 48, or 72 h after infection. The concentrations of IFN-α and IFN-β in the culture supernatants were assessed with sandwich ELISA kits (PBL Biomedical Laboratories, Piscataway, NJ), according to the manufacturer’s instructions.

### Viral RNA extraction and reverse transcription (RT)-PCR

JAWS II cells in 12-well culture plates (5 × 10^5^ cells/well) were infected with CVS or ERA at an MOI of 10. Following incubation for 48, 72, 96, or 120 h, the cells were collected and washed three times with PBS. The total RNA was then extracted from the cells using the acid-guanidinium thiocyanate -phenol -chloroform method (TRIzol®, Gibco BRL, Gaithersburg, MD). To detect the viral genomic RNA (negative polarity) or viral mRNA amplification, a partial sequence of the N gene was amplified using the sense primer NF2850 (nucleotides 28–50, 5′-ACAGACAGCGTCAATGGCAGAGC-3′) and antisense primer N660 (R) (nucleotides 660–676, 5′-GTTTGGTATAGTACTCC-3′) (Nishizono et al. 2002). The primer positions are given according to the N gene of CVS (GenBank accession number DQ286762). Total RNA (1 μg) was reverse transcribed to synthesize the first-strand cDNA of the viral genome RNA using the primer N660 (R) or the viral mRNA using the primer NF2850 and Moloney murine leukemia virus reverse transcriptase (Gibco BRL) at 37°C for 2 h. The first PCR reaction was performed with the following reaction mixture: 1 μg of sample cDNA, 50 mM KCl, 10 mM Tris–HCl (pH 8.4), 1.5 mM MgCl_2_, 20 mM each of primers NF2850 and N660 (R), 200 μM dNTPs, and 2 U of *Taq* DNA polymerase (Promega Corp., Madison, WI). The reaction mixture was subjected to 35 cycles of denaturation at 95°C for 30 s, annealing at 50°C for 30 s, and extension at 72°C for 90 s. The resulting amplicons were resolved electrophoresed on 1% agarose gels and stained with ethidium bromide. As the internal control mRNA, glyceraldehyde phosphate dehydrogenase (GAPDH) transcripts were amplified under the same conditions, with the sense primer 5′-TTCACCACCATGGAGAAGGC-3′ and the antisense primer 5′-GGCATGGACTGTGGTCATGA-3′.

### Effect of naïve spleen cells on RABV-infected JAWS II cells

JAWS II cells were seeded in six-well culture plates (10^6^ cells/well) and infected with CVS or ERA at an MOI of 10.The cells were collected after 48h incubation, washed three times in PBS, and then plated in a new six-well culture plate (10^5^ cells/well). Spleen cells were obtained from the spleens of normal C57BL/6 mice (Otsu et al. 2006) (Charles River Japan Inc., Yokohama, Japan) and then cultured with or without the RABV-infected JAWS II cells (10^6^ cells/well; final E/T ratio = 10:1). After 120 h of coculture, the cells were observed microscopically (Olympus CK30, Olympus, Tokyo, Japan) and a cytotoxicity assay was performed with the Cyto Tox 96® Non-Radioactive Cytotoxicity Assay (Promega Corp.). Cytotoxicity was calculated according to the formula: % cytotoxicity = (Experimental LDH release - Effector Spontaneous LDH release - Target Spontaneous LDH release / Target Maximum LDH release - Target Spontaneous LDH release × 100).

### Cell-to-cell transmission of RABV from JAWS II cells to neural cells *in vitro*

JAWS II cells or NA cells in six-well culture plates (10^6^ cells/well) were infected with CVS at an MOI of 10. After incubation for 48 h at 37°C, the cells were collected and washed thoroughly three times with PBS. A virus titration assay was performed to confirm that there were no remaining viruses in the supernatant, as described above. The cells were then added to a 24-well plate containing monolayers of cultured NA cells. After 48 h of coculture, the NA cells were stained with FITC-conjugated anti-RABV N protein MAb and observed under a laser scanning microscope.

### Injection of CVS- or ERA-inoculated JAWS II cells into mouse brains

JAWS II cells in six-well culture plates (10^6^ cells/well) were infected with CVS or ERA at an MOI of 30. Following incubation for 48 h, the cells were collected and washed three times with PBS. A virus titration assay was performed to confirm that no viruses remained in the supernatant, as described above. Specific pathogen-free five-week-old female C57BL/6 mice (Seac Yoshitomi, Fukuoka, Japan) were injected intracerebrally with 10^6^, 10^5^, or 10^4^ CVS- or ERA-infected JAWS II cells in 0.03 mL with a 23-gauge needle. Half the mice in each injected group (n = 10) were observed for 2 weeks and their survival was recorded daily. The remaining mice were sacrificed on day 7 and the tissue samples taken from the hippocampus were analyzed for viral N protein using a laser scanning microscope, for viral mRNA and an immunochromatographic test that had been developed previously by us (Nishizono et al. 2008). All animal procedures conformed to animal care guidelines approved by Ethics Committee in Oita University.

## Authors’ information

Kazuyo Senba: DDS, PhD

Kagoshima University Graduate School of Medical and Dental Sciences

Area of specialization: Virology, Immunology, Bacteriology

Takashi Matsumoto: PhD

Oita University Graduate School of Medical Sciences

Area of specialization: Virology, Immunology, Bacteriology

Kentaro Yamada: DVM, PhD

United Graduate School of Veterinary Sciences

Area of specialization: Virology

Seiji Shiota: MD, PhD

Oita University Graduate School of Medical Sciences

Area of specialization: Immunology,

Hidekatsu Iha: PhD

The Graduate University for Advanced Studies

Area of specialization: Cell biology

Yukari Date: MD, PhD

Miyazaki University Graduate School of Medical Sciences

Area of specialization: Biochemistry

Motoaki Ohtsubo: PhD

Graduate School of Medical Sciences, Kyushu University

Area of specialization: Cell biology

Akira Nishizono: MD, PhD

Oita University Graduate School of Medical Sciences

Area of specialization: Virology, Immunology, Bacteriology

## Authors’ original submitted files for images

Below are the links to the authors’ original submitted files for images. Authors’ original file for figure 1 Authors’ original file for figure 2 Authors’ original file for figure 3 Authors’ original file for figure 4 Authors’ original file for figure 5 Authors’ original file for figure 6

## Abbreviations

DC: Dendritic cells
MHC: Major histocompatibility complex
IFN: Interferon
RIG 1: Retinoic-acid-inducible gene I
RABV: Rabies virus
CNS: Central nervous system
ERA: Evelyn-Rokitnicki-Abelseth
FACS: Fluorescence activated cell sorting
TLR: Toll-like recepetor
HIV: Human immunodeficiency virus
MDC: Myeloid-type DC
HEV: High endothelial venules
FITC: Fluorescein isothiocyanate
PE: Phycoerythrin
MAb: Monoclonal antibody
GADPH: Glyceraldehyde phosphate dehydrogenase.
