# Supplementary figures and images for: Passive carriage of rabies virus by dendritic cells
Source: Springerplus. 2013 Aug 29;2(1):419. doi: 10.1186/2193-1801-2-419 (PMC3765594; doi:10.1186/2193-1801-2-419)

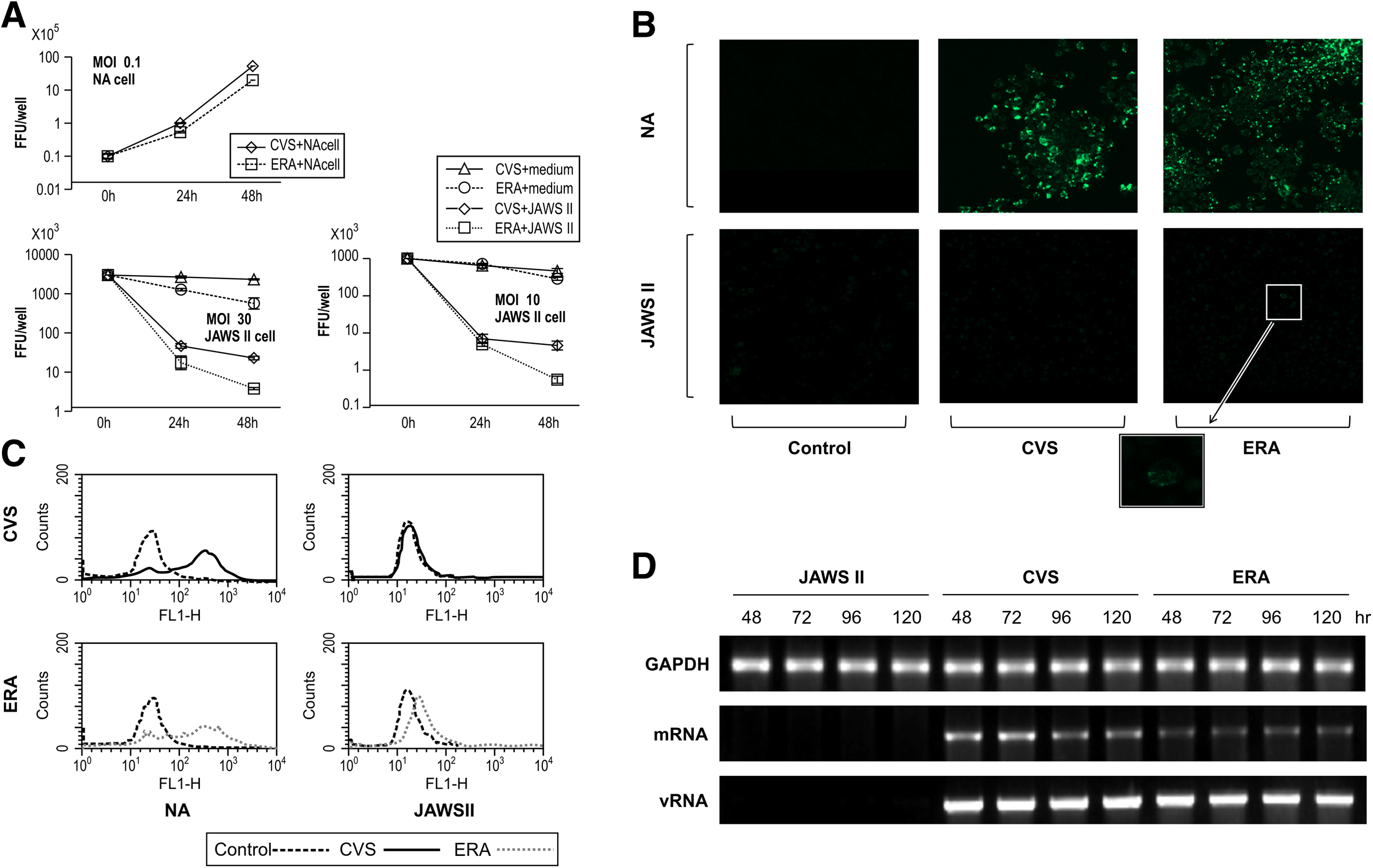

Supplement: Supplementary file 1 — Authors’ original file for figure 1 [file 40064_2013_483_MOESM1_ESM.tif]

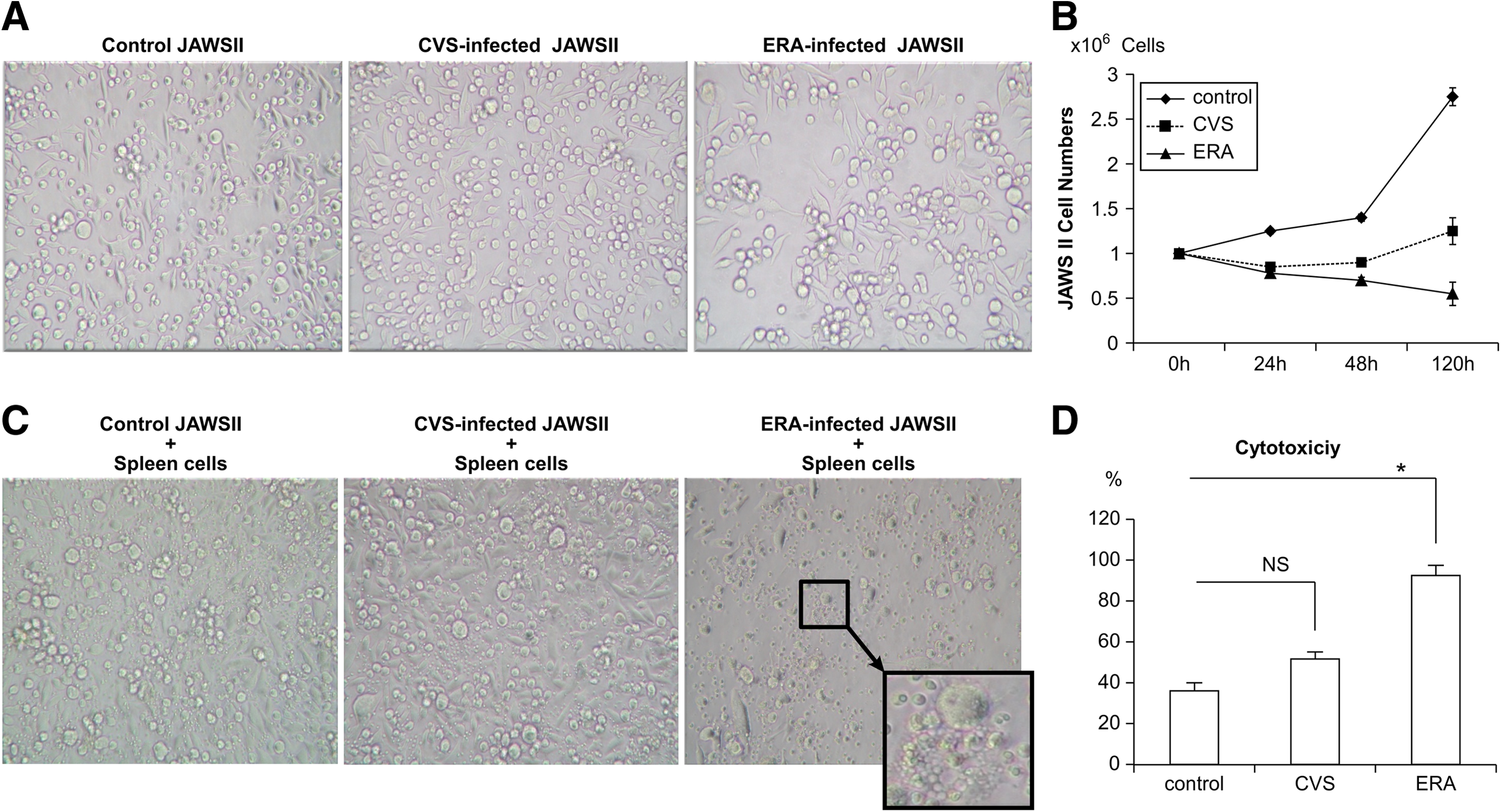

Supplement: Supplementary file 2 — Authors’ original file for figure 2 [file 40064_2013_483_MOESM2_ESM.tif]

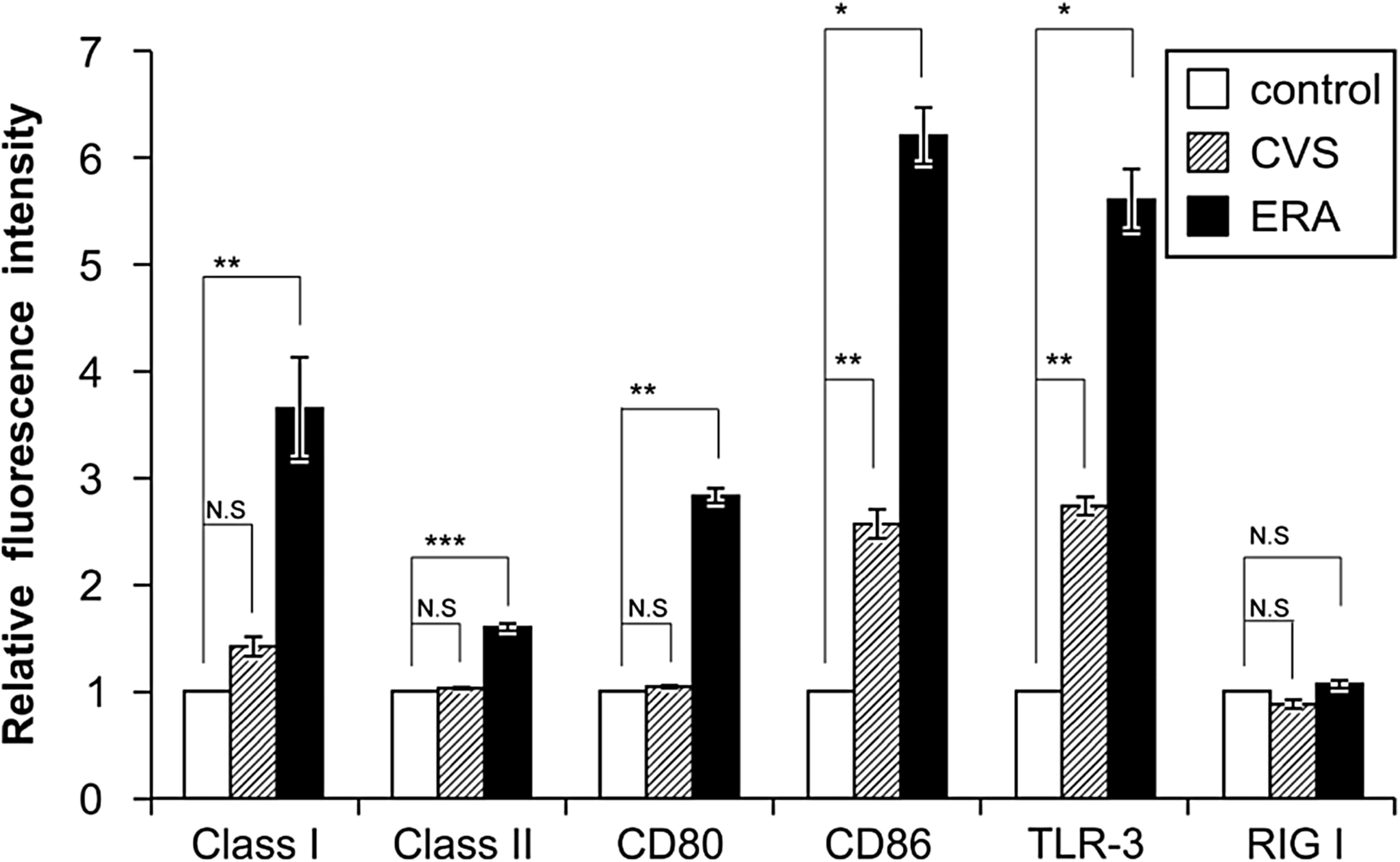

Supplement: Supplementary file 3 — Authors’ original file for figure 3 [file 40064_2013_483_MOESM3_ESM.tif]

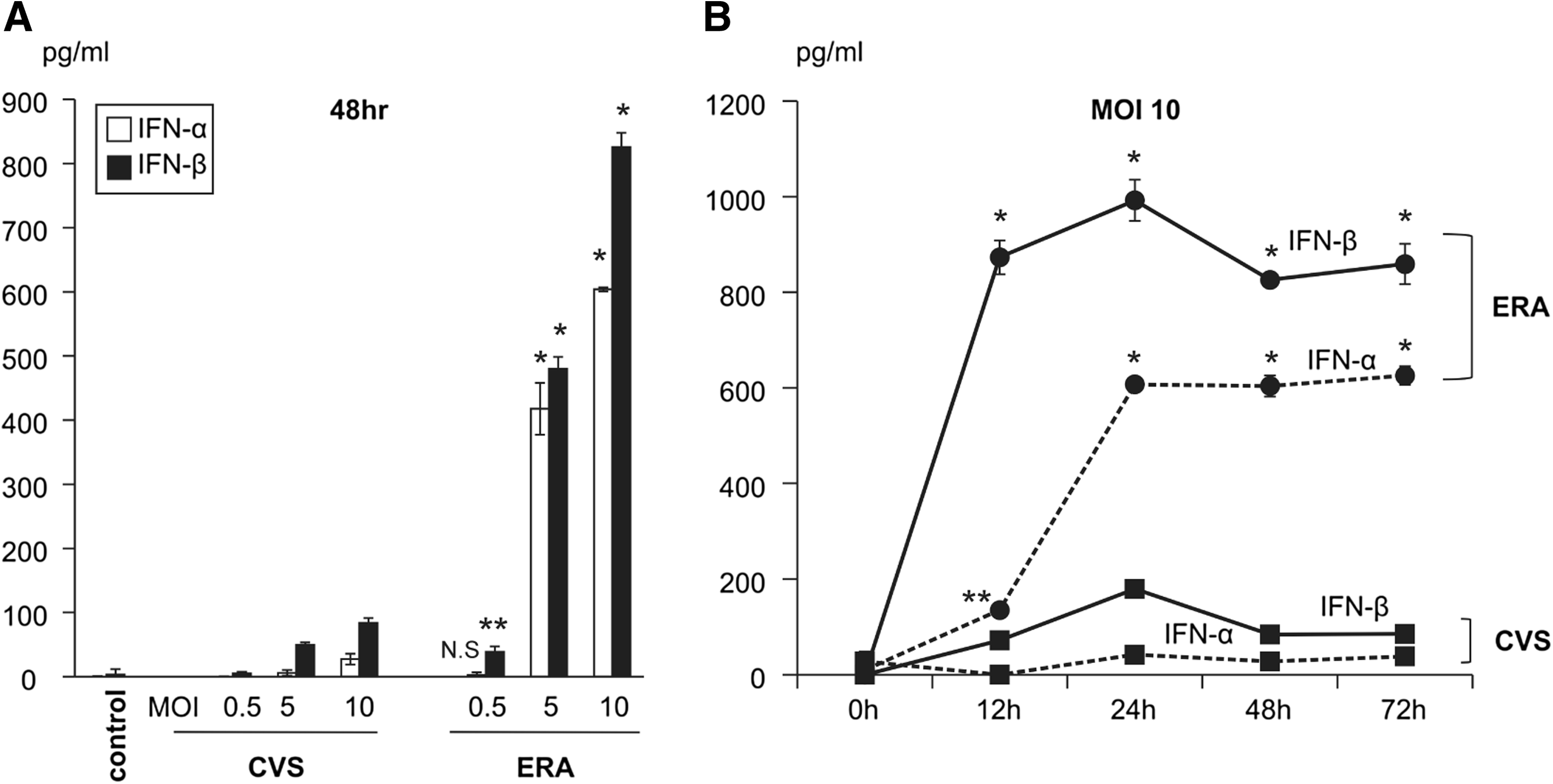

Supplement: Supplementary file 4 — Authors’ original file for figure 4 [file 40064_2013_483_MOESM4_ESM.tif]

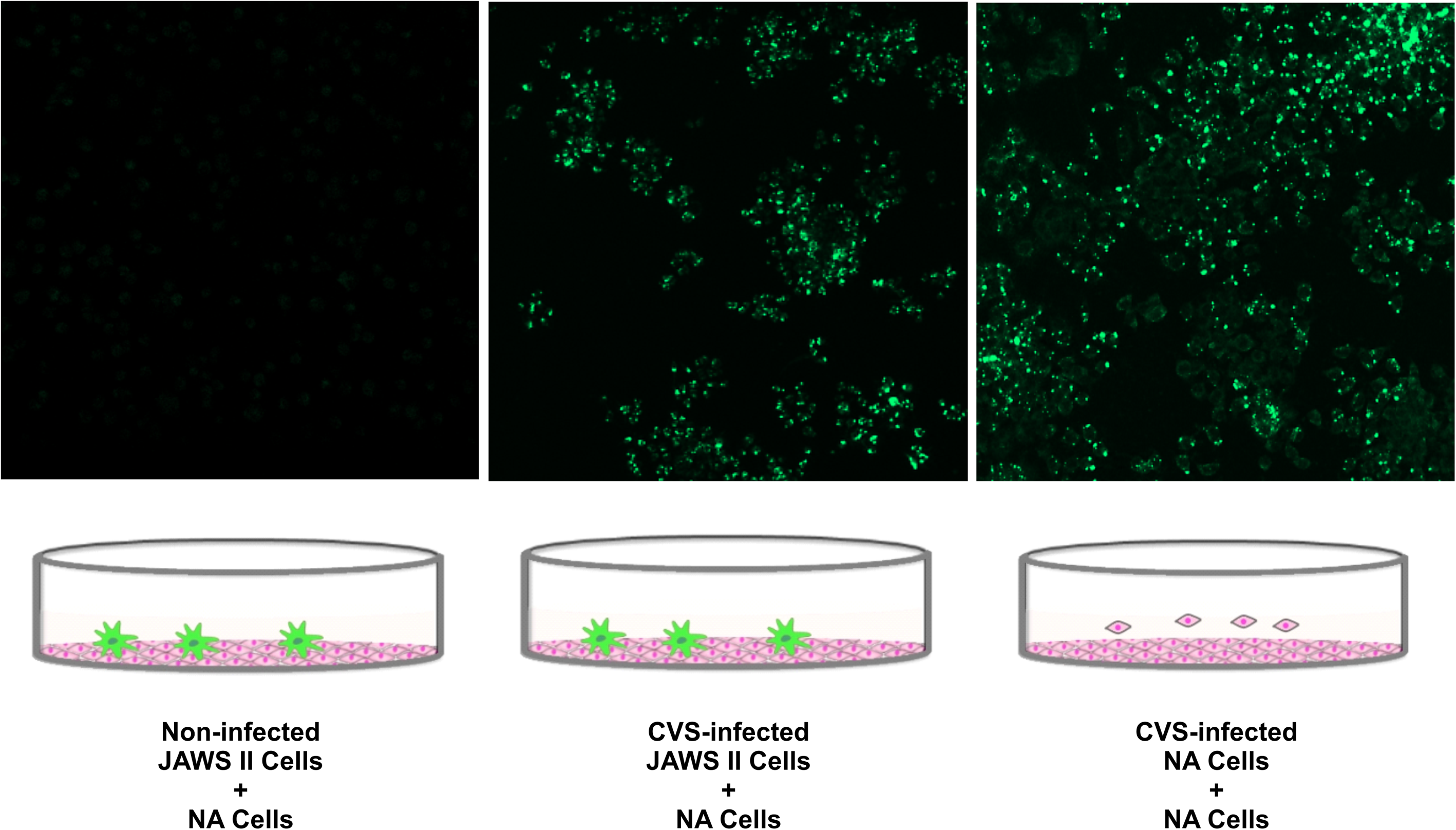

Supplement: Supplementary file 5 — Authors’ original file for figure 5 [file 40064_2013_483_MOESM5_ESM.tif]

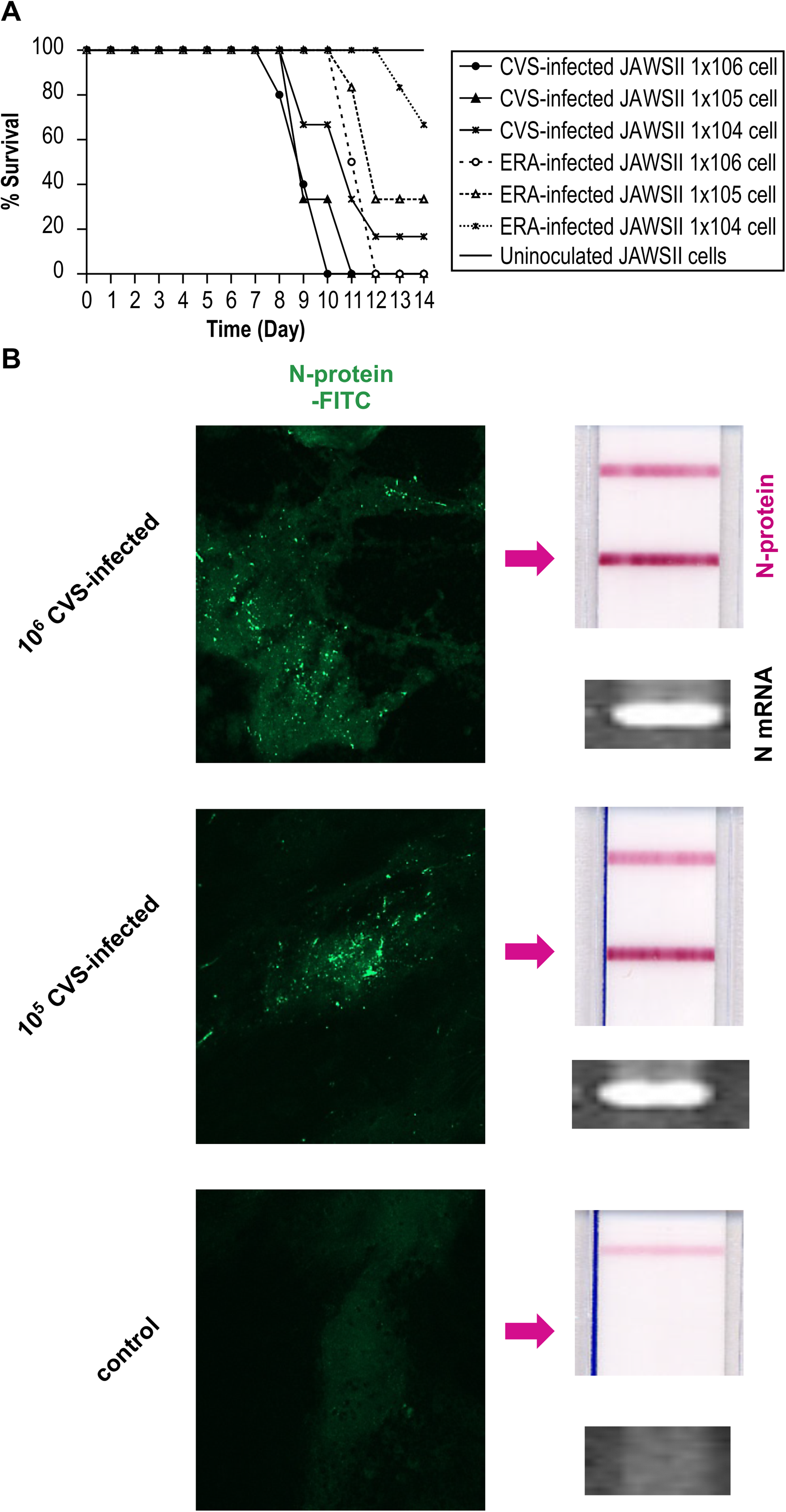

Supplement: Supplementary file 6 — Authors’ original file for figure 6 [file 40064_2013_483_MOESM6_ESM.tif]
